# Ectopic Sebaceous Glands in the Hair Follicle Matrix: Case Reports and Literature Review of this Embryogenic Anomaly

**DOI:** 10.7759/cureus.3605

**Published:** 2018-11-17

**Authors:** Lizy M Paniagua Gonzalez, Jaime A Tschen, Philip R Cohen

**Affiliations:** 1 Internal Medicine, University of Texas Medical Branch, Galveston, USA; 2 Dermatology, St. Joseph Dermatopathology, Houston, USA; 3 Dermatology, San Diego Family Dermatology, San Diego, USA

**Keywords:** anomaly, developmental, ectopic, embryogenic, follicle, gland, hair, matrix, sebaceous

## Abstract

Hair embryogenesis is a complex process. The development of this skin appendage originates from both ectoderm and mesoderm layers. Multiple signaling pathways and regulation are required for proper hair formation. However, anomalies occasionally arise, such as ectopic sebaceous glands in the hair follicle matrix. Two men who demonstrate this developmental anomaly are reported and the characteristics of individuals in whom this aberration in hair follicle maturation has occurred are reviewed. In addition, the anatomy of the hair follicle is summarized and the embryologic features of hair morphogenesis are discussed. The occurrence of hair follicle matrix ectopic sebaceous glands is an observation of intellectual intrigue for which the pathogenesis and clinical implications remain to be determined.

## Introduction

Ectopic sebaceous glands within the hair follicle matrix are rarely observed. To the best of our knowledge, this phenomenon has only been described once [1]. We report two men who also demonstrated this developmental anomaly and review the characteristics of individuals in whom this has occurred.

## Case presentation

Case 1

A 30-year-old Caucasian man presented for evaluation of a pigmented lesion on the left side of his chin. He requested the removal of the dark lesion for cosmetic reasons. A cutaneous examination showed a 2 x 2 millimeter brown papule.

**Figure 1:**
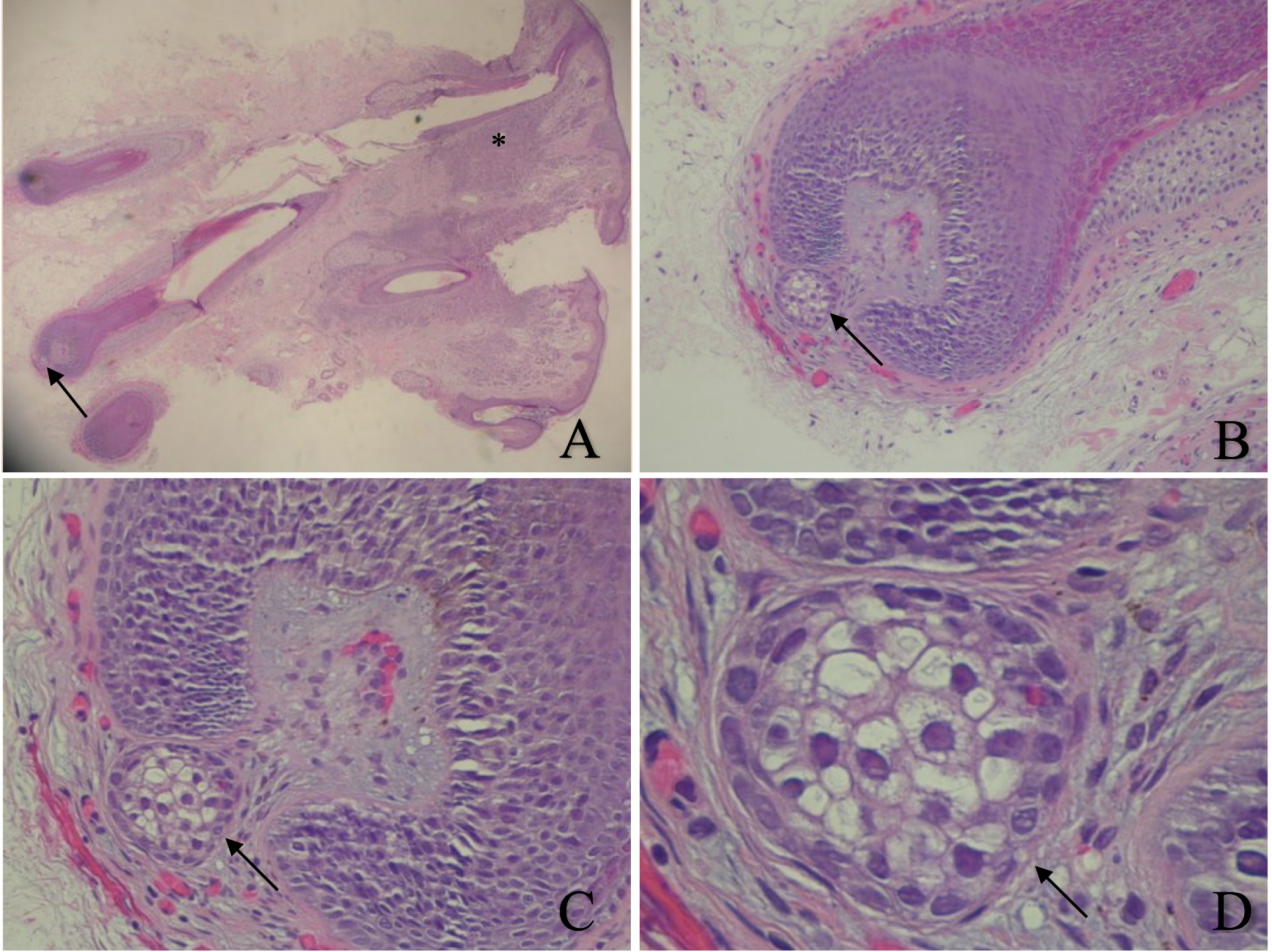
Ectopic sebaceous gland in the hair papilla. Low (A) and higher (B, C, and D) views of a sebaceous gland (arrow) that is located in the hair papilla of a compound nevus—with benign-appearing nests of melanocytes in the dermis (asterisk) and along the basal layer of the epidermis—that was located on the left side of the chin of a 30-year-old man (Hematoxylin and eosin: A, x20; B, x40; C, x200; D, x400).

A 4 millimeter punch excision was performed. Microscopic examination revealed benign-appearing nests of melanocytes in the dermis and along the basal layer of the epidermis, establishing the diagnosis of a compound nevus. In addition, a prominent hair follicle showed a small sebaceous gland in the hair follicle papilla (Figure 1).

Case 2

A 30-year-old Caucasian man presented for evaluation of multiple, small 2-3 millimeter papules on his face. His dermatologic history is significant for erythromelanosis follicularis faciei—an uncommon sporadic pigmentary disease of undetermined etiology characterized by follicular papules and erythematous hyperpigmented patches on the face. A papule on his chin was biopsied.

**Figure 2:**
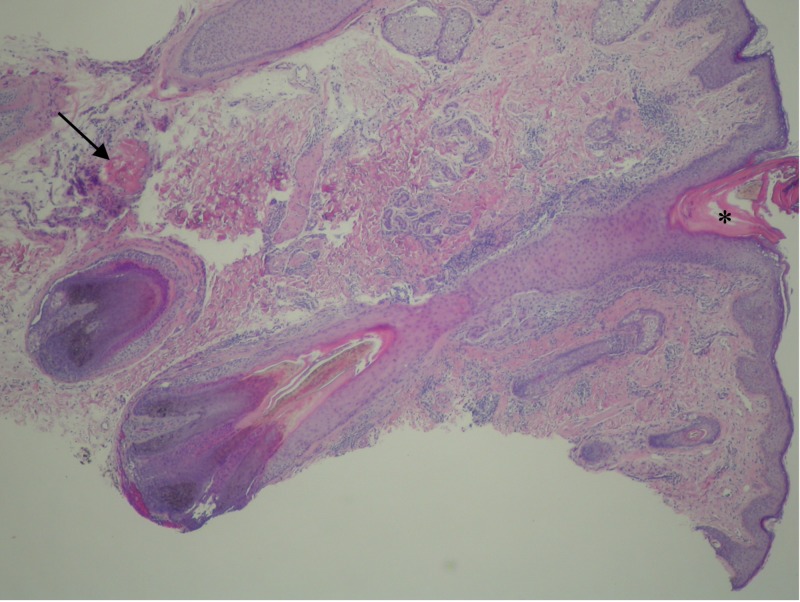
Keratosis pilaris and pilomatricoma. Compact keratin (asterisk) plugs the follicular orifice of a hair follicle; this is consistent with keratosis pilaris. In the deep reticular dermis, there is a small keratinocytic tumor containing shadow cells (arrow); this is a pilomatricoma (Hematoxylin and eosin: x 20).

**Figure 3:**
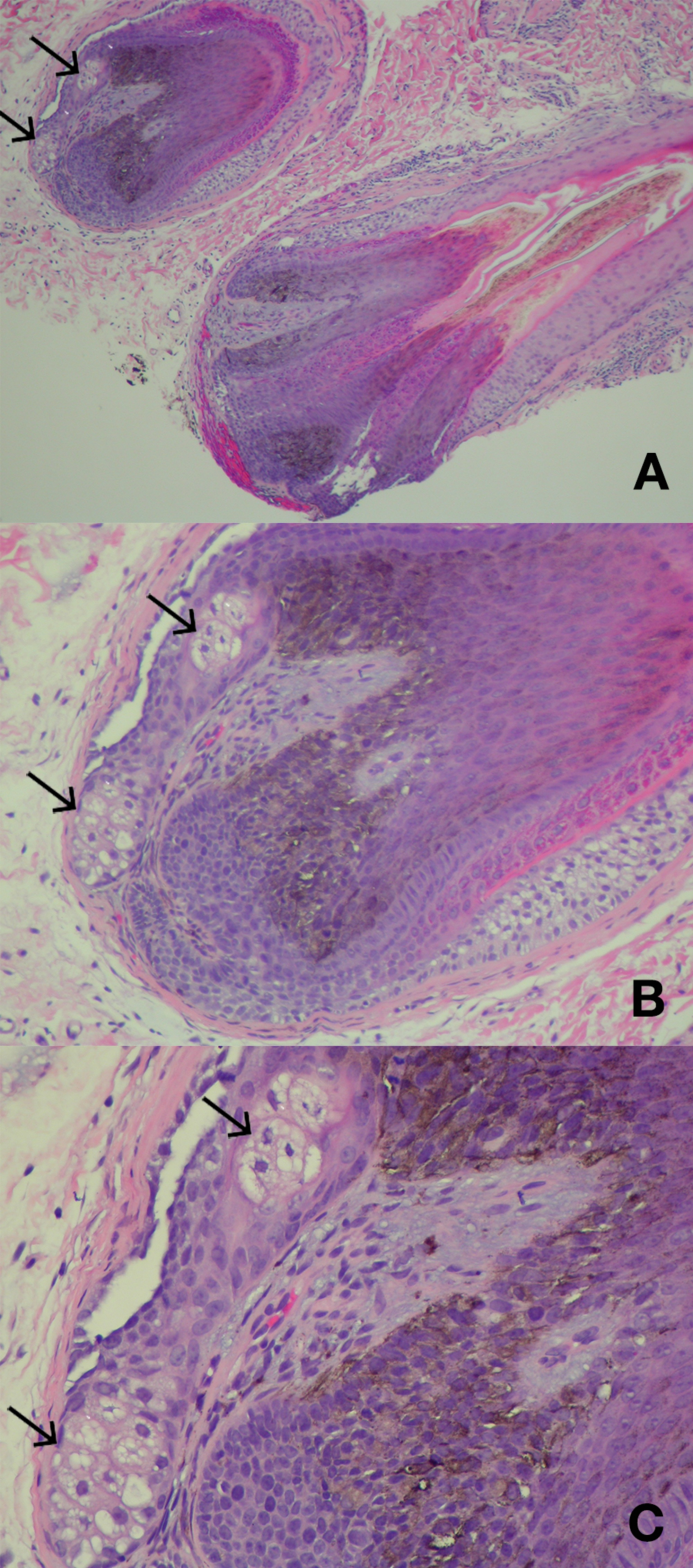
Ectopic sebaceous glands in hair papilla. Low (A) and higher (B and C) views of ectopic glands (arrows) seen within the follicular bulbs of hair follicles from the chin of a 30-year-old man (Hematoxylin and eosin: A, x20; B, x40; C, x200).

Microscopic examination showed follicular plugging and a small keratinocytic dermal tumor containing shadow cells; these findings were consistent with keratosis pilaris and a pilomatricoma (Figure 2). In addition, ectopic sebaceous glands were seen within multiple hair follicle papillae (Figure 3).

## Discussion

Hair is composed of differentiated keratinocytes; they are referred to as keratin. The hair follicle is divided into four compartments: the infundibulum, the isthmus, the suprabulbar, and the bulb. Within these compartments, the hair follicle contains several structures: the hair matrix, the dermal papilla, the outer and inner root sheath, the bulge, and the pilosebaceous unit [2].

The pilosebaceous unit is made of sebaceous glands, the apocrine gland, and the arrector pili muscle. This unit is located in the area demarcated by the hair follicle infundibulum and isthmus. The sebaceous duct drains into the infundibulum of the hair follicle and the sebaceous gland is located superior to the arrector pili muscle bulge [2,3].

The bulge is the area where the epithelial and melanocytic hair follicle stem cells are localized. The outer root sheath is the area into which the erector pili muscle inserts. The bulb is where the dermal papillae, hair melanocytes, and the hair matrix are localized.

Most of the hair follicle cells are from ectoderm and mesoderm origins. The hair follicle stem cells, sebaceous glands, apocrine gland originate from the ectoderm during embryogenesis. In contrast, the dermal papillae, and the inner and outer sheath develop from the mesoderm [2,3].

Hair follicle embryogenesis is a complex interaction and communication between the epidermis and the dermis [4]. Hair development starts around day 14 of embryogenesis, with the formation of dermal papilla. First the placode, which is composed of epidermal thickening, forms; then the cells send a signal that leads to the development of the dermal condensate underneath [4-6]. Communication between the placode and the dermal condensate results not only in the proliferation of epithelial placode cells but also their downward extension; eventually the placode envelopes the dermal condensate (which is now termed the dermal papilla) and becomes the hair bulb [4-7].

The dermal papilla is a type of mesenchymal cell. It regulates the growth and the development of the hair follicle during embryogenesis and postnatal life [4-6]. The dermal papilla signals the adjacent epithelial cells--called matrix cells--to proliferate and differentiate.

The dermal papilla cells become the inner root sheath. They also develop into the hair shaft layers as they move upward during maturation [5,6]. The outer root sheath layer surrounds the inner root sheath; it is continuous with the interfollicular epithelial cells. The bulge is a region of the outer root sheath that contains epithelial stem cells [7].

Sebocytes originate from superficial hair follicle cells; the cells expand and form glands [7]. Sebaceous gland development occurs between the 13th-16th week of gestation from the superficial bulge of the emerging hair follicle [8]. Sonic hedgehog (Shh), Wnt signaling pathway, heparan sulfate protein, and C-myc have been implicated in the development, regulation, and proliferation of the sebocyte cells [3,7-9].

The hair follicle signaling and developmental pathways have been elucidated [2,4-6,10-13]. Therefore, it is possible that the presence of ectopic sebaceous glands within the hair matrix may be due to a disruption in the hair development process.

The finding of a sebaceous gland within the hair matrix is a rare developmental anomaly. Tschen et al. reported the first individual with this abnormality in the Journal of Cutaneous Pathology in 2006 [1]. The patient was a 21-year-old man with a five year history of a recalcitrant perioral folliculitis which was made worse by shaving. The condition did not improve after multiple treatments including mupirocin 2% cream, sulfacetamide 10% wash, azelaic acid 15% cream, minocycline 100 milligrams daily (initially for five months and then three additional six-week courses), valacyclovir 500 milligrams daily, and levofloxacin 500 milligrams daily for two weeks. His cutaneous examination showed perioral, 3-5 millimeter, papulopustules. A potassium hydroxide preparation did not show either yeast or Demodex mites, excluding folliculitis secondary to either Candida yeast or Demodex mites. Normal skin flora was grown from the bacterial cultures from his nostril and a skin pustule. Therefore, he underwent two biopsies on his chin; an ectopic sebaceous gland and duct was located within the bulb of an anagen hair follicle from his right chin biopsy [1].

The clinical features of the three Caucasian men with ectopic sebaceous glands in their hair follicle matrix are summarized in Table 1. They ranged in age from 21 years to 30 years at when their skin lesions were biopsied; the mean age was 27 years and the median age was 30 years. The lesions had been present for a minimum of five years; the facial papules for two of the men had been present since childhood.

**Table 1:** Clinical features of patients with hair follicle matrix ectopic sebaceous glands ^a^Abbreviations:  C, case; mm, millimeters; Ref, references; yrs, years.

C	Age Race Gender	Onset	Lesion site	Size (mm)	Morphology	Skin condition	Ref
1	21 yrs, White, Male	16 yrs old	Chin	3x3 to 5x5	Papulopustule	Recalcitrant perioral folliculitis	[1]
2	30 yrs, White, Male	Childhood	Chin	2x2	Brown papule	Compound nevus	Current report Case 1
3	30 yrs, White, Male	Childhood	Chin	2x2 to 3x3	Flesh-colored papule	Erythromelanosis follicularis faciei Pilomatricoma	Current report Case 2

Therefore, the affected hairs were on the face. specifically, they were located on the chin; the significance of this interesting observation remains to be determined. The clinical presentation of the hair-containing lesion was a papule—either flesh-colored, brown, or papulopustular. The lesion was small, ranging in size from 2 x 2 millimeters to 5 x5 millimeters.

Two of the men had facial dermatoses. One man had an acneiform perioral folliculitis that was recalcitrant to therapy. Another man had erythromelanosis follicularis faciei, a condition that presents with follicular facial papules and reddish to brownish patches on the face; his biopsy showed keratosis pilaris-like changes (follicular plugging) and a small pilomatricoma. The third man had a benign compound nevus.

All of the men demonstrated one or more normal appearing sebaceous glands ectopically located within the matrix portion of one or more hair follicles. In addition, the chin biopsy from the 21-year-old man who had a perioral dermatosis also showed a sebaceous duct in the hair follicle matrix.

## Conclusions

Multiple signaling and developmental pathways influence the morphogenesis of hair. Sebaceous glands are normally located in the mid portion of the hair follicle between the infundibulum and the isthmus. However, albeit rare, ectopic sebaceous glands located within the hair bulb have been observed. Specifically, ectopic sebaceous glands in the hair papilla that occurred in three young men were observed; the affected facial hairs were on their chin. The associated clinical scenario varied; for one of the men, the ectopic sebaceous gland was an incidental finding in a benign compound nevus. The other men had underlying dermatoses: either a therapy-resistant perioral folliculitis or erythromelanosis follicularis faciei. The pathogenesis of ectopic sebaceous glands in the hair follicle matrix remains to be established. Also, it is intriguing to consider whether these ectopic sebaceous glands that are found in the hair follicle matrix serve a specific function. In summary, additional observations of this developmental anomaly may provide additional insight into the embryogenesis and pathophysiology of hair follicle maturation and disorders.
